# Mid-Regional Pro-Adrenomedullin Is Associated with Adverse Cardiovascular Outcomes After Cardiac Surgery

**DOI:** 10.3390/jpm15020047

**Published:** 2025-01-26

**Authors:** Ulrike Baumer, Niema Kazem, Andreas Hammer, Felix Hofer, Eva Steinacher, Lorenz Koller, Daniel Zimpfer, Martin Andreas, Barbara Steinlechner, Christian Hengstenberg, Alexander Niessner, Patrick Sulzgruber

**Affiliations:** 1 Division of Cardiology, Department of Internal Medicine II, Medical University of Vienna, 1090 Vienna, Austria; 2 Division of Cardiac Surgery, Department of Surgery, Medical University of Vienna, 1090 Vienna, Austria; 3 Division of Cardiothoracic Anesthesiology and Intensive Care, Department of Anesthesiology, Intensive Care and Pain Medicine, Medical University of Vienna, 1090 Vienna, Austria; 4 2nd Department of Medicine with Cardiology and Intensive Care Medicine, Vienna Healthcare Group, Clinic Landstrasse, Medical University of Vienna, 1030 Vienna, Austria

**Keywords:** heart failure, MR-proADM, cardiac surgery, biomarker

## Abstract

**Background:** In the era of personalized medicine, tools for risk stratification after cardiovascular interventions are crucial to reduce mortality and morbidity, especially in the aging population. Biomarker-based approaches, in particular, have gained significant importance. Mid-regional pro-adrenomedullin (MR-proADM) represents an easily assessable biomarker that mirrors cardiac function and fibrosis. Therefore, we aimed to investigate the prognostic potential of MR-proADM in patients undergoing elective cardiac surgery. **Methods:** Patients undergoing elective cardiac bypass and/or valve surgery were prospectively enrolled between May 2013 and August 2018. The primary endpoint was the composite of hospitalization for heart failure (HHF) or cardiovascular (CV) mortality. **Results:** In total, 500 patients (146 female [29.2%]; median age 69.8 years (IQR 60.6–75.5 years) were included. Individuals were stratified into risk categories based on their MR-proADM values (Low Risk ≤ 0.63 nmol/L, Intermediate Risk > 0.63 and ≤0.84, High Risk > 0.84). A significant increase in 5-year event rates for HHF/CV mortality in patients in the high-risk category (Low Risk 8.6% vs. High Risk 37.7%, *p* < 0.001) was observed. MR-pro ADM showed an independent association with HHF/ CV mortality (adjusted HR of 3.43, 95% CI 1.83–6.42; *p* < 0.001 comparing the High-Risk group to the Low-Risk group). **Conclusions:** MR-pro ADM was found to be a strong and independent predictor for HHF/CV mortality in patients undergoing elective cardiac surgery. Considering a personalized diagnostic and prognostic work-up, a standardized preoperative evaluation of MR-proADM levels might help to identify patients at risk for major adverse events and early re-hospitalization.

## 1. Introduction

Considering the aging population, mortality and morbidity following cardiac surgery continue to be significant challenges to achieving favorable long-term outcomes. Despite substantial advancements in protecting perioperative myocardial function, medical therapy, and postoperative care, heart failure (HF) and mortality due to disease progression remain prevalent during the postoperative period. Although complication and mortality rates are declining, adverse events such as sepsis, bleeding, ischemic events, HF, and postoperative atrial fibrillation (POAF) continue to impact patient outcomes [1]. In the era of personalized medicine and tailored prognostic assessments, identifying predictive markers offers a compelling approach to risk stratification in clinical settings. Consequently, discovering new biomarkers that reflect various facets of the underlying pathophysiological process could enhance diagnostic assessment and offer supplementary prognostic insights. Additionally, to enhance risk assessment before surgery, there is a growing utilization of biomarker-based approaches to appraise the postoperative prognosis that patients encounter [2,3].

Adrenomedullin is a regulatory peptide expressed in most tissues of the human body, including the heart, lungs, kidneys, and endocrine and blood cells. Initially most acknowledged due to its vasodilatory function, it also influences cellular growth and differentiation and has been proven to be associated with cardiovascular (CV) disorders [4,5]. Recent evidence has shown that MR-proADM has strong and independent potential as both a diagnostic and prognostic biomarker for HF [6,7,8,9]. Elevated levels of MR-proADM are associated with increased mortality and rehospitalization in both chronic and acute HF [10,11]. Furthermore, MR-proADM levels rise with increasing NYHA classes, reflecting disease severity [12].

Considering the available data in the literature and the importance of a personalized diagnostic and prognostic preoperative work-up, MR-proADM might be a promising biomarker for risk stratification before the index surgical procedure in patients scheduled for elective CABG and/ or valve replacement. Therefore, we aimed to investigate the prognostic value of a standardized preoperative assessment of MR-proADM levels on HHF and CV mortality in patients undergoing elective cardiac surgery.

## 2. Materials and Methods

### 2.1. Study Population

**Figure 1:**
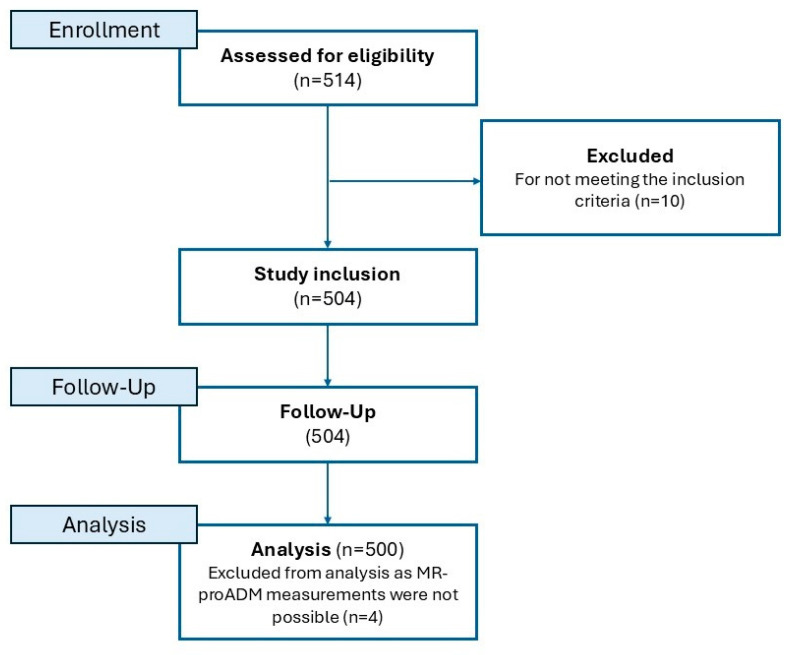
Study flow chart.

In this prospective observational study, 500 patients undergoing elective valve and/or bypass surgery were enrolled. All surgical interventions were conducted at the Department of Cardiac Surgery at the Medical University of Vienna (Austria) between May 2013 and August 2018. All patients admitted for elective coronary artery bypass graft (CABG) and/ or valve surgery were eligible. Exclusion criteria were age of ≤18 years, atrial fibrillation at hospital admission or within the last 6 months before admission, non-elective cardiac surgery, and planned percutaneous or transapical valve implantation. Participants were enrolled at the time of hospital admission prior to surgery and gave written informed consent. At the time of hospital admission, an ECG was assessed to confirm the patients’ sinus rhythm since a history of AF was a criterion for non-eligibility. The study process is illustrated in Figure 1. The study protocol adheres to the principles of the Declaration of Helsinki and has received approval from the Ethics Committee at the Medical University of Vienna (No. 1110/2013).

### 2.2. Data Acquisition and MR-proADM Measures

Patients’ demographics were assessed using a standardized questionnaire, including their medical history and clinical presentation. Routine laboratory blood samples were processed by the Department of Laboratory Medicine of the General Hospital of Vienna (Medical University of Vienna). For MR-proADM measures, a preoperative, peripheral venous blood sample was taken at the time of enrollment. The blood samples were centrifuged at 3000 bpm for 20 min at 4 °C (Allegra X-12R Centrifuge; Beckman Coulter, United States). The harvested plasma was subsequently stored at −80 °C for further analysis. MR-pro ADM levels were measured using an automated immunofluorescence assay (B B·R·A·H·M·S MR-proADM KRPYTOR), with a detection limit of 0.05 nmol/L, a limit of quantitation of 0.23 nmol/L, and an inter-assay coefficient of variability of 20% at 0.25 nmol/L.

### 2.3. Follow-Up and Endpoints

The primary outcome was the composite of CV death or HHF. Secondary outcomes were the individual components of the primary endpoint and POAF and all-cause mortality. For the primary endpoint, the cause of death was acquired via the national death registry, and for HHF, data from the Vienna Healthcare Group hospitalizations database (“Wiener Gesundheitsverbund”) were assessed.

Postoperatively, patients were monitored and screened for the emergence of POAF. During the early postoperative period (within 9 days after surgery), patients were continuously observed using a 3-lead surface electrocardiographic system. In the event of an arrhythmic episode, POAF was documented using a 12-lead surface electrocardiogram for over 30 s, in line with recent guidelines of the European Society of Cardiology [13].

### 2.4. Statistical Analysis

Categorical data were illustrated as counts and proportions and analyzed using the Chi-Square Test. Continuous variables were shown as medians with the Inter-Quartile Range (IQR) and analyzed using the Kruskal–Wallis test. To determine MR-proADM risk groups for CV mortality, a Classification And Regression Tree (CART) analysis was performed. Observation periods were assessed using the Kaplan–Meier estimator, and event rates for the individual endpoints were compared using the log-rank test. To assess the risk for the primary and secondary endpoints with increasing MR-proADM values, a Cox regression model was used. The multivariate model was adjusted for age, sex, type of surgery, diabetes mellitus, chronic obstructive pulmonary disease (COPD), N-terminal prohormone of brain natriuretic peptide (NT-pro BNP), Creatinine and C-reactive protein (CRP). A two-sided *p*-value of <0.05 was considered statistically significant. All calculations were performed using SPSS (version 29.0.0.0) and R (version 4.0.4; R Foundation for Statistical Computing, Vienna, Austria).

## 3. Results

### 3.1. Study Population

A total of 500 patients were enrolled in the present study. Participants were followed prospectively over a median of 4.6 years (IQR 3.0–5.8). The median age was 69.8 years, and 146 (29.2%) were female. Within the entire study population, 214 (42.8%) individuals underwent valve surgery, 160 (32.0%) coronary artery bypass graft (CABG) surgery, and 126 (25.2%) patients received a combined (CABG and valve replacement) procedure.

CART analysis revealed a cut-off-value for MR-proADM of ≤0.63 nmol/L (=Low Risk) 0.64–0.84 nmol/L (=Intermediate Risk) and ≥0.84 nmol/L (=High Risk). Detailed characteristics of the entire study population and those stratified into risk categories are illustrated in Table 1. As expected, patients in the highest risk group tended to be older, had a lower left ventricular ejection fraction, and presented with higher overall morbidity (illustrated by the CHA2DS2-VASc score and EuroSCORE II, respectively, *p*-value < 0.001). In higher-risk groups, patients were more likely to receive a combined procedure (CABG and valve replacement, *p*-value 0.008).

### 3.2. Impact of MR-proADM on Cardiovascular Outcomes

After a median follow-up time of 4.6 years (IQR 3.0–5.8), 83 patients (16.6%) reached the predefined composite primary endpoint. We observed that MR-proADM was strong and independently associated with HHF/CV death with an adjusted HR of 1.58 (95%CI: 1.28–1.96; *p*-value < 0.001). A consistent association was evident for the secondary endpoints CV death (adjusted HR of 1.70; 95% CI: 1.34–2.14, *p*-value < 0.001) and all-cause mortality (adjusted HR of 1.78; 95% CI: 1.43–2.22, *p*-value < 0.001).

**Figure 2:**
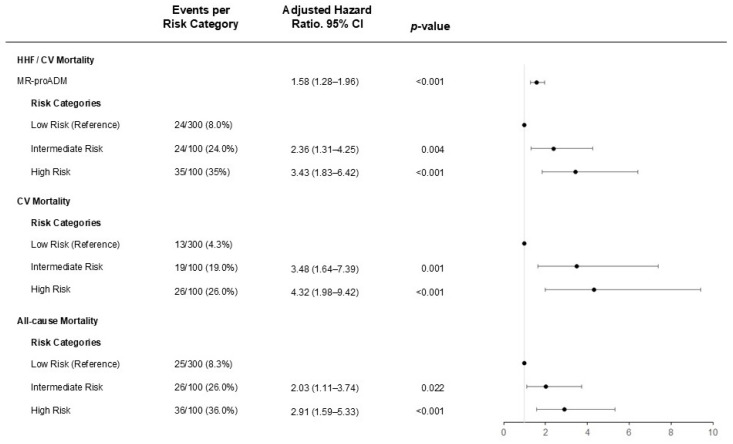
Relationship between MR-proADM and CV outcomes. Risk groups for CV mortality were analyzed via CART analysis with Low Risk: MR-proADM ≤ 0.63 nmol/L, Intermediate Risk: MR-proADM > 0.63 nmol/L and ≤0.84 nmol/L, High Risk: MR-proADM > 0.84 nmol/L. Patients in the MR-proADM Low-Risk group were used as references. The Cox regression model has been adjusted for age, sex, type of surgery, diabetes mellitus, COPD, NT-pro BNP, Creatinine, and C-reactive protein. HHF = hospitalization for heart failure, CV = cardiovascular.

**Figure 3:**
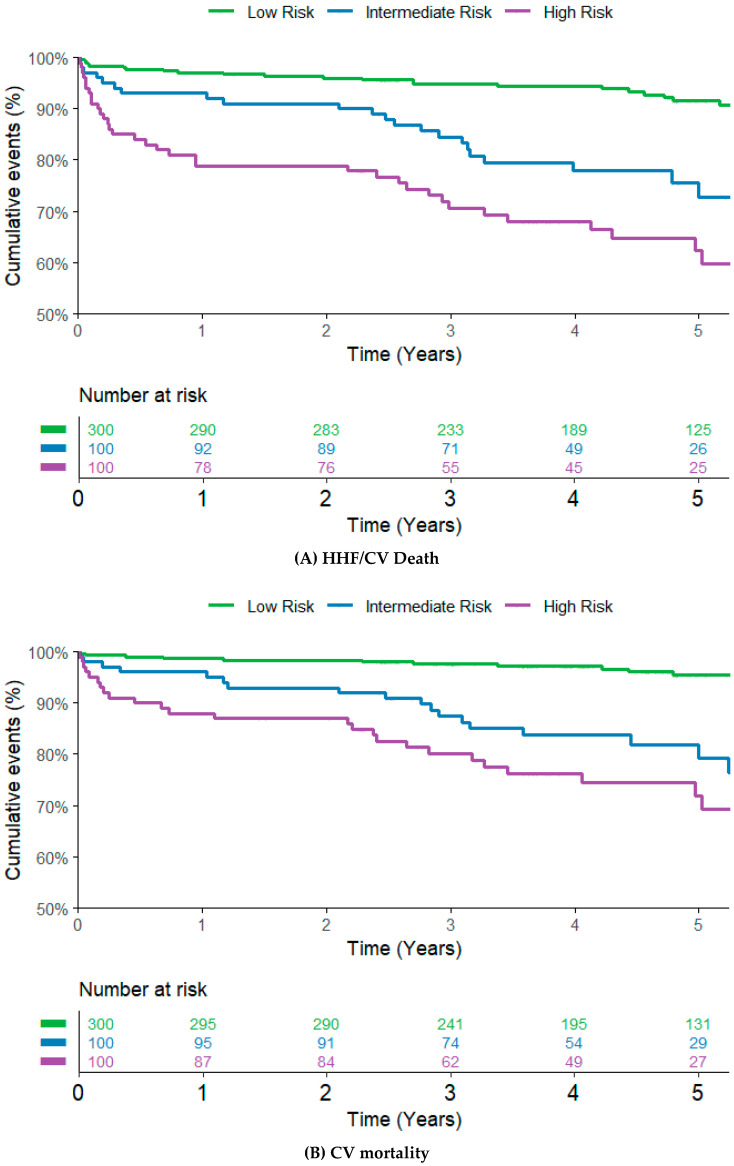

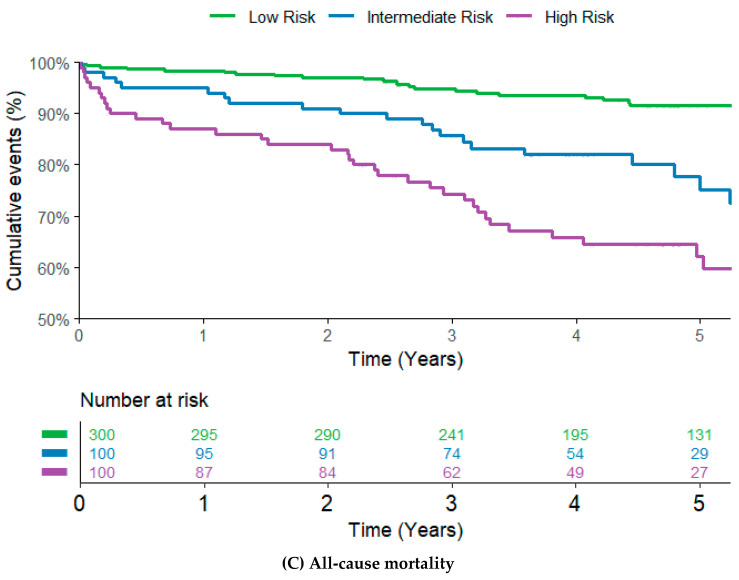
Kaplan–Meier curves and the corresponding 5-year Kaplan–Meier event rates for (**A**) the composite of HHF or CV death, (**B**) CV mortality, and (**C**) All-cause mortality stratified by MR-proADM Risk groups. Kaplan–Meier Curve with 5-year event rates of 8.6% in the Low-Risk group, 27.3% in the Intermediate Risk group, and 37.7% in the High-Risk group, *p* < 0.001. Kaplan–Meier Curve with 5-year event rates of 4.6% in the Low-Risk group, 20.8% in the Intermediate-Risk group, and 28.2% in the High-Risk group, *p* < 0.001. Kaplan–Meier Curve with 5-year event rates of 8.5% in the Low-Risk group, 24.8% in the Intermediate-Risk group, and 37.8% in the High-Risk group, *p* < 0.001.

Notably, a concentration-dependent effect of MR-proADM was observed, illustrating a more than three-folded increase in risk comparing the Low- to the High-Risk category (adjusted HR of 3.43, 95% CI 1.83–6.42, *p*-value < 0.001) for the primary endpoint. (Figure 2). The findings were confirmed and graphically illustrated by Kaplan–Meier survival plots (see Figure 3). The Receiver-Operating Characteristic (ROC) curve showed similar performance for NT-proBNP, with an area under the curve (AUC) of 0.729 (95% CI 0.671–0.786), compared to an area under the curve of 0.727 (95% CI 0.669–0.785) for MR-proADM regarding the primary endpoint HHF/CV mortality. Reclassification analysis showed a net reclassification index of 0.42 (95% CI 0.18–0.65, *p*-value 0.0004) and an integrated discrimination increment of 0.01 (95% CI −0.001–0.023, *p*-value 0.077). A risk assessment including both EuroScore and CHA_2_DS_2_VASc score is displayed in Table 2.

### 3.3. Association of MR-proADM and Postoperative Atrial Fibrillation

A total of 211 patients (42.2%) developed POAF during their hospital stay. There was a significant association between the occurrence of POAF and MR-proADM levels with an OR of 1.70 (95% CI 1.13–2.55, *p*-value 0.010).

Analysis of the previously assessed risk groups revealed a significant increase in the risk of developing POAF in the High-Risk group compared to the Low-Risk group, with an OR of 2.02 (95% CI 1.28–3.20, *p* = 0.003), as summarized in Table 3.

## 4. Discussion

To the best of our knowledge, the present investigation represents the first and largest analysis demonstrating that MR-proADM is independently associated with the composite endpoint HHF/ CV death, as well as CV death, all-cause mortality, and POAF in an unselected patient cohort undergoing elective cardiac surgery. Moreover, MR-proADM showed promising diagnostic capability in identifying high-risk patients who may require in-depth clinical attention, as illustrated in Figure 2. Therefore, MR-proADM, as a novel biomarker, could complement and enhance the utility of existing markers. Its integration within a personalized and tailored risk stratification approach might significantly improve the prevention of adverse cardiovascular outcomes in individuals undergoing cardiac surgery.

### 4.1. MR-proADM as Prognostic Biomarker for Cardiovascular Outcomes

Adrenomedullin is a regulatory peptide, particularly known for its powerful vasodilatory effect. While lowering blood pressure, it increases blood flow, preserves endothelial integrity, and is an inhibitor of the renin–angiotensin–aldosterone system [14]. In the general population, elevated MR-proADM levels have been found to be associated with higher rates of CV and all-cause mortality [15]. Furthermore, it has been established as a biomarker in septic patients, providing prognostic information on the severity of the disease as well as the short- and long-term survival [16]. In the Australia–New Zealand Heart Failure Study, MR-proADM was independently associated with both mortality and HHF in 297 patients with chronic ischemic left ventricular dysfunction [17]. In our patient population, we observed a more than three-fold increased risk for the composite of HHF/ CV death in patients with high MR-proADM values, indicating MR-proADM as an independent prognostic factor in long-term morbidity and mortality. When compared to NT-proBNP, we could observe a comparable AUC, and even after adjusting for other risk scores, such as the EuroSCORE and the CHA_2_DS_2_VASc score, there was still a nearly three-fold increase in risk for the primary endpoint in the highest-risk group.

### 4.2. Postoperative Atrial Fibrillation

POAF is one of the most common adverse events after cardiac surgery, affecting up to 40% of patients. Its pathogenesis is multifactorial, influenced by various risk factors, including local inflammation and atrial remodeling [18]. Previously, associations have been identified between POAF and several novel biomarkers, including midregional pro-atrial natriuretic peptide, galectin-3, growth differentiation factor-15, and fibroblast growth factor-23 [19,20,21,22]. In the general population, there seems to be a significant trend between the development of AF and higher MR-proADM levels [23]. Moreover, in patients undergoing pulmonary vein radiofrequency ablation, higher baseline MR-proADM levels have indicated a higher risk of AF recurrence [24]. Our data showed a significant association between higher MR-proADM values and the development of POAF, indicated by an OR of 1.70 (95% CI 1.13–2.55, *p*-value 0.010) after comprehensive adjustment. Given that POAF can progress to long-term AF, resulting in increased short- and long-term mortality rates and a higher risk of stroke, preoperative individual risk assessment is crucial [18]. Incorporating MR-proADM into risk assessment for individuals undergoing cardiac surgery could thereby provide a valuable prognostic tool for POAF and its associated adverse events.

### 4.3. Risk Assessment in Cardiac Surgery

As minimally invasive procedures are on the rise, preoperative risk assessment is vitally important to enable the best possible patient-oriented therapy decision [25]. A study by Schoe et al. in 2015 involving patients undergoing elective cardiac procedures demonstrated that postoperative MR-proADM values are a feasible tool for predicting intrahospital mortality [26]. In patients with vasodilatory shock after cardiac surgery, significantly higher MR-proADM levels were observed compared to patients with uncomplicated postoperative course by Hillinger et al. in 2022 [27]. Moreover, MR-proADM has been proven to be a prognostic marker not only in conventional cardiac surgery but also in minimally invasive operations. Particularly, in a study of 100 patients undergoing transcatheter aortic valve implantation (TAVI), higher preoperative levels of MR-proADM were associated with an increased risk of adverse events [28]. Our data further supports the prognostic impact of preoperative MR-proADM measurements in patients undergoing cardiac surgery.

### 4.4. Clinical Implementation and Personalized Risk Assessment

Adrenomedullin levels will likely mirror the cardiac and systemic response to cardiac impairment. In addition to its potent vasodilatory action, adrenomedullin enhances contractility through multiple pathways, including Ca^2+^ release and increased Ca^2+^ influx [29].

In the present study, CART analysis enabled the stratification of risk groups for adverse events based on patients’ baseline MR-proADM values. Patients with preoperative MR-proADM levels of >0.84 nmol/L showed an increase in risk for HHF/CV death and a twofold chance of developing POAF post-procedure compared to patients in the Low-Risk group. It is well known that POAF generally results in an increased incidence of adverse events and worse clinical outcomes [18]. Identifying these POAF-susceptible patients allows for implementing preventive measures, including pharmaceutical and interventional treatments [30,31]. Moreover, the onset of HF ranks among the leading causes of hospital readmission after cardiac surgery [32]. The preoperative assessment of MR-proADM effectively identified high-risk patients, enabling the possible implementation of proactive management strategies. Both CV and all-cause mortality were shown to be significantly associated with higher MR-proADM levels with a 3- to 4-fold increase in risk in patients with values of >0.84 nmol/L.

Within the present study, MR-proADM successfully identified vulnerable patients. Therefore, incorporating this marker into personalized preoperative risk prediction assessments might be suggested, as it may allow physicians to evaluate the net clinical benefit of the procedure and implement preventive strategies for adverse events.

### 4.5. Limitations

Some limitations regarding this study warrant consideration. Firstly, this is a single-center study. Therefore, generalization to the wide public may not be possible. Regarding HHF, there might be the possibility of underreporting due to recall bias. Finally, despite multiple adjustments, residual confounding is still possible.

## 5. Conclusions

MR-pro ADM was found to be a strong and independent predictor for HHF/CV mortality in an unselected cohort undergoing elective cardiac surgery. Considering a personalized diagnostic and prognostic preoperative work-up, a standardized preoperative evaluation of MR-proADM levels might help to identify patients at risk for major adverse events and early re-hospitalization.

## Figures and Tables

**Table 1:** Baseline characteristics.

Variable	All Patients (*n* = 500)	Low Risk (*n* = 300)	Intermediate Risk (*n* = 100)	High Risk (*n* = 100)	*p*-Value
**Clinical Presentation**					
Age, years (median, IQR)	70 (61–76)	67 (58–24)	73 (66–78)	72 (66–78)	<0.001
Sex, female, (*n*, %)	146 (29.2)	82 (27.3)	31 (31.0)	33 (33.0)	0.506
Body mass index, kg/m^2^ (median, IQR)	27.2 (24.3–30.2)	26.8 (24.1–29.7))	27.7 (24.9–30.9)	28.2 (24.3–30.9)	0.095
Type of surgery					
Valve replacement (*n*, %)	214 (42.8)	130 (43.3)	43 (43.0)	41 (41.0)	0.919
CABG, (*n*, %)	160 (32.0)	109 (36.3)	26 (26.0)	25 (25.0)	0.039
Valve replacement and CABG, (*n*, %)	126 (25.2)	61 (20.3)	31 (31.0)	34 (34.0)	0.008
NYHA Class, (*n*, %)					0.059
I	26 (9)	15 (9.6)	8 (12.5)	3 (4.4)	
II	125 (43.3)	79 (50.3)	24 (37.5)	22 (32.4)	
III	120 (41.5)	55 (35.0)	28 (43.8)	37 (54.4)	
IV	18 (6.2)	8 (5.1)	4 (6.3)	6 (8.8)	
LVEF	60 (54.2–60)	60 (60–60)	60 (50–60)	55 (40–60)	<0.001
Left atrial Diameter, mm (median, IQR)	55 (45–65)	55 (45–60)	55 (45–65)	60 (55–65)	<0.001
CHA_2_DS_2_VASc score (median, IQR)	4 (3–5)	3 (2–4)	4 (3–5)	4 (4–5)	<0.001
EuroSCORE II (median, IQR)	2.3 (1.2–4.5)	1.7 (1.0–2.8)	2.7 (1.6–4.8)	6.7 (3.2–12.2)	<0.001
**Comorbidities (*n*, %)**					
Coronary artery disease	302 (60.4)	178 (59.3)	61 (61.0)	63 (63.0)	0.802
Previous MCI	130 (26.0)	69 (23.0)	28 (28.0)	33 (33.0)	0.125
Hypertension	405 (81.0)	234 (78.0)	84 (84.0)	87 (87.0)	0.096
Diabetes mellitus	144 (28.8)	69 (23.0)	31 (31.0)	44 (44.0)	<0.001
Heart failure	289 (57.8)	157 (52.3)	64 (64)	68 (68)	0.009
COPD	69 (13.8)	30 (10)	14 (14)	25 (25)	<0.001
Current smoker	61 (12.2)	38 (12.7)	10 (10.0)	13 (13.0)	0.751
**Laboratory measures at admission**					
MR-proADM nmol/L (median, IQR)	0.58 (0.44–0.79)	0.48 (0.36–0.56)	0.73 (0.67–0.79)	1.12 (0.94–1.43)	<0.001
NT-proBNP, pg/mL (median, IQR)	539.6 (209.4–1455.0)	324.3 (140.5–694.3)	573.2 (241.7–1289.0)	2116.0 (957.1–6062.0)	<0.001
C-reactive protein, mg/dL (median, IQR)	0.2 (0.1–0.5)	0.2 (0.1–0.4)	0.2 (0.1–0.5)	0.4 (0.2–1.1)	<0.001
Creatinine, mg/dL (median, IQR)	0.95 (0.8–1.2)	0.87 (0.75–1.0)	1.04 (0.91–1.18)	1.39 (1.16–1.87)	<0.001

Baseline characteristics by MR-proADM risk groups with Low Risk: MR-proADM ≤ 0.6335 nmol/L, Intermediate Risk: MR-proADM > 0.6335 and ≤0.8382, High Risk: MR-proADM > 0.8382; IQR = Inter-Quartile Range, CABG = Coronary Artery Bypass Graft, NYHA = New York Heart Association, LVEF = Left ventricular ejection fraction, MCI = Myocardial Infarction, COPD = Chronic Obstructive Pulmonary Disease, NT-proBNP = N-terminal prohormone of brain natriuretic peptide.

**Table 2:** Hazard ratios for the primary and secondary endpoints adjusted for the EuroScore and CHA_2_DS_2_VASc score.

	HR	95% CI	*p*-Value
**HHF/CV Mortality**			
MR-proADM	1.77	1.23–2.55	**0.002**
Intermediate Risk	2.34	1.30–4.24	**0.005**
High Risk	2.76	1.52–5.02	**<0.001**
**CV Mortality**			
MR-proADM	2.11	1.42–3.13	**<0.001**
Intermediate Risk	3.63	1.76–7.49	**<0.001**
High Risk	3.87	1.90–7.85	**<0.001**
**All-cause mortality**			
MRpro-ADM	2.23	1.63–3.07	**<0.001**
Intermediate Risk	2.22	1.24–3.97	**0.007**
High Risk	2.89	1.67–4.97	**<0.001**

Risk groups were analyzed via CART analysis with Low Risk: MR-proADM ≤ 0.63 nmol/L, Intermediate Risk: MR-proADM > 0.63 nmol/L and ≤0.84 nmol/L, High Risk: MR-proADM > 0.84 nmol/L. Patients in the MR-proADM Low-Risk group were used as references. The Cox regression model has been adjusted for EuroScore and CHA_2_DS_2_VASc score. HHF = hospitalization for heart failure, CV = cardiovascular.

**Table 3:** Odds Ratios for POAF.

POAF	OR	95% CI	*p*-Value
MR-proADM	1.70	1.13–2.55	**0.010**
Intermediate Risk	1.25	0.79–1.98	0.344
High Risk	2.02	1.28–3.20	**0.003**

Risk groups were analyzed via CART analysis with Low Risk: MR-proADM ≤ 0.63 nmol/L, Intermediate Risk: MR-proADM > 0.63 nmol/L and ≤0.84 nmol/L, High Risk: MR-proADM > 0.84 nmol/L. Odds Ratios were assessed compared to the Low-Risk group; OR = Odds Ratio, POAF = postoperative atrial fibrillation.

## Data Availability

Data are available upon reasonable request.

## Abbreviations

The following abbreviations are used in this manuscript:

CABG	Coronary artery bypass graft
CART	Classification And Regression Tree
CRP	C-reactive protein
CV	Cardiovascular
HF	Heart failure
HHF	Hospitalization for heart failure
IQR	Interquartile range
MR-proADM	Mid-regional pro adrenomedullin
NT-pro BNP	N-terminal prohormone of brain natriuretic peptide
POAF	Postoperative atrial fibrillation
